# 筛查发现的小体积非小细胞肺癌治疗相关的问题探讨——术中定位、切除范围、淋巴结清扫

**DOI:** 10.3779/j.issn.1009-3419.2016.06.09

**Published:** 2016-06-20

**Authors:** 德若 刘, 真榕 张

**Affiliations:** 100029 北京，中日友好医院胸外科 Department of Thoracic Surgery, China Japan Friendship Hospital, Beijing 100029, China

**Keywords:** 肺肿瘤, 筛查, 肺叶切除, 亚肺叶切除, 淋巴结清扫, Lung neoplasms, Lung screening, Lobectomy, Sublobectomy, Lymph node resection

## Abstract

随着胸部计算机断层扫描（computed tomography, CT）的广泛应用，以及肺癌高危人群筛查的逐渐开展，越来越多的“小肺癌”被发现及诊断。“小肺癌”具有的术中定位困难、侵袭性弱、近期和远期预后较好的特点为肺癌的外科治疗提出了新的挑战，本文着重从肺癌的筛查、小结节定位、肺叶切除与亚肺叶切除以及淋巴结清扫范围等方面对“小肺癌”带来的挑战进行探讨。

## Abstract (en)

With the widely application of chest computed tomography (CT) and gradual generalization of lung cancer screening project for high risk populations, more and more "tiny lung cancers" were identified. Due to the difficulty in intraoperative orientation, their less aggressiveness, and excellent short/long term prognosis, the 'tiny lung cancers' posed new challenges for the surgery in the treatment of lung cancer. In this review, theirfore, we focused on these challenges and presented the progress in lung cancer screening, orientation of lung nodules, lobectomy and sublobectomy, and lymph node dissection.

肺癌是目前世界上发病率与死亡率最高的恶性肿瘤，其中男性肺癌发病率和死亡率均占所有恶性肿瘤的第一位，女性发病率占第二位，死亡率占第二位。每年在世界范围内，因肺癌死亡的人口达137, 000, 000，占所有癌症死亡比例的18%^[1]^。我国资料显示2015年中国预计有429.2万例新发肿瘤病例和281.4万例死亡病例，肺癌占其比例17.1%，死亡率高达21.1%。肺癌目前已成为威胁中国人生命健康的“头号杀手”^[2]^。随着我国老龄人口的增加，城乡工业化进程加快，以及环境污染的加剧，肺癌的发病率和死亡率都将会持续上升，因此肺癌的预防和早期发现将成为我国肿瘤控制的最为重要的一环。

以外科治疗为主的综合治疗是早期非小细胞肺癌（non-small cell lung cancer, NSCLC）的主要治疗模式。自从1933年Evarts A Graham第一次报道成功实施左全肺切除治疗中心型肺癌，肺癌外科治疗已经经历了70余年的发展。肺癌外科治疗的标准术式也由全肺切除+纵隔淋巴结清扫逐渐发展为肺叶切除+纵隔淋巴结清扫。在过去的几十年里，超过2/3的肺癌患者因在发现时已经发生远处转移而丧失手术根治的机会。然而随着胸部电子计算机断层扫描（computed tomography, CT）的广泛应用，以及肺癌高危人群筛查的逐渐开展，越来越多的“小肺癌”被发现及诊断。这不仅改变了不同病理类型肺癌所占的比例，即腺癌（40%）已经取代鳞癌成为NSCLC的主要病理类型^[3]^；也使早期肺癌比例逐年升高，使得更多的患者获得了根治性手术以及长期生存的机会。同时由于“小肺癌”具有体积小、术中难于发现、肿瘤侵袭性较弱、肿瘤预后相对较好的特点，这为肺癌的外科治疗提出了新的挑战。

## 1 肺癌筛查

肺癌常规筛查手段包括胸部X线、支气管镜、痰检、血清和其它体液的生化标志检查等，但这些筛查方式受敏感度及特异度限制。常规CT虽然敏感度及特异度相对较高，但累积辐射剂量较大，且费用昂贵。因此如何在辐射剂量与诊断图像质量之间找到最佳平衡点是近年来国内外研究热点之一。20世纪90年代以来，低剂量CT（low dose CT, LDCT）作为一种新的检查手段应运而生。与常规胸片相比，CT较为敏感。早期肺癌行动计划研究发现，CT所发现的Ⅰ期肺癌是胸片的6倍。其次CT检查无创、快捷，目前技术条件下，患者屏息数秒即可完成胸部扫描，放射剂量仅为3.3毫伏，相当于一个人全年自然环境放射总量。不仅如此，随着技术进步CT检查的放射剂量有可能进一步降低^[4]^。

2002年-2004年美国肿瘤协会开展的多中心临床试验（国家肺筛选试验）对超过53, 000个有重度吸烟史（每年至少吸30包烟）的无症状患者进行长期（3年以上）随访^[5, 6]^。该研究将病例随机分入LDCT组或胸片组。结果显示与胸片组相比，LDCT能够发现更多的肺癌患者，且多数（50%）为Ⅰa期患者；LDCT可以使肺癌死亡的绝对风险下降0.33%，相对风险下降20%。因此国家肺筛选试验得出年龄在55岁-74岁之间、有重度吸烟史（> 30包/年）的男性和女性人群可以从LDCT筛查中获益。基于国家肺筛选试验令人兴奋的结果，美国国家肺癌协作网络肺癌筛查委员会、美国胸内科医师协会、美国临床肿瘤协会以及美国肺脏协会均推荐对一定的肺癌高危人群进行LDCT筛查。基于已发表的非随机对照研究和观察性研究的数据，2015年2月5日美国卫生监管部门批准LDCT肺癌筛查进入医保覆盖范围。

近年来，我国越来越多的医疗机构已开展或拟开展LDCT肺癌筛查，但尚未形成具体的肺癌筛查指导意见，并缺乏相应的诊疗规范。我们所面临的最大挑战是如何基于国外肺癌筛查经验，谨慎而深思熟虑地开展肺癌筛查，使更多高危人群从筛查中获益，降低肺癌筛查给患者及社会所带来的不必要的心里以及社会经济负担。

## 2 肺小结节定位

影像学表现为“磨玻璃影”特点的“小肺癌”的增多为胸外科技术提出了新的挑战。病灶越小（< 10 mm）、病变实性成分比例越低（< 50%）、距离肺表面越远（> 5 mm），术中的准确定位率越低。以往靠手术医生手指的触觉来寻找病灶的位置具有一定的局限性。胸腔镜肺组织活检中，外科医生只能肉眼定位或手指触及约45%的肺部结节，一些患者因无法找到病灶而被迫中转开胸。因此一些胸外科、呼吸科以及放射科医生开始探索如何术前准确定位病变，为术中切除病变所在部位，同时尽可能保护肺组织，并有效降低诊断性开胸提供依据。良好的术前定位方法应具有创伤小、操作简单、定位精确、不易移位的特点，能够起到降低诊断性开胸或胸腔镜解剖性切除周围型肺小结节的比率，缩短胸腔镜肺切除手术时间并且不增加临床相关并发症的作用。

目前报道可用于术前辅助定位这些结节的技术，包括染料定位、硬化剂定位、CT引导下螺旋线定位、术中超声、放射性核素成像等。由于操作简便易行，染料定位、硬化剂定位以及CT引导下螺旋线定位等方法是目前临床应用最多的定位方法。然而其各自局限性限制了这些方法的普及程度：染料定位容易扩散，影响手术切除的精确性；硬化剂定位增加了术后病理诊断的难度；弹簧圈定位法气胸发生率及压缩程度较高；术中超声扫描对于肺气肿病人敏感性过低。近年来近红外成像定位技术、无创CT引导下肺标记技术、电磁导航支气管镜技术等新型术前辅助定位技术均取得了令人满意的前期结果，但仍需要更多的临床数据证实其安全性及有效性。

## 3 肺叶切除和亚肺叶切除

解剖性肺叶切除联合淋巴结清扫一直是早期肺癌的标准术式。随着辅助检查技术的发展，越来越多的早期肺癌得到及时诊断。正电子发射计算机断层显像（positron emission tomography/CT, PET/CT）的临床应用提高了术前分期的准确性，有助于肺癌的早期诊断；肺癌的CT筛查研究结果证实了CT筛查有助于早期肺癌的发现，广泛应用的CT技术使得很多直径 < 1 cm的早期肺癌被发现。因此肺叶切除作为早期肺癌外科治疗的金标准的传统观念受到了挑战。基于亚肺叶切除具有对呼吸功能影响较小、手术技术成熟、术后并发症发生率低、术后恢复快的特点，外科医生开始探索早期或多原发肺癌是否可以通过亚肺叶切除获得与肺叶切除相媲美的肿瘤治疗效果。

1996年美国肺癌研究组（Lung Cancer Study Group, LCSG）研究^[7]^，该研究将最大径 < 3 cm的早期NSCLC患者随机分为肺叶切除组和亚肺叶切除组（包括楔形切除和肺段切除），研究结果显示亚肺叶切除组的局部复发率为肺叶切除组的3倍，远期生存也劣于肺叶切除组。然而由于该研究纳入患者肿瘤体积较大、术前并未常规进行PET/CT、纵隔镜检查以及并未对亚肺叶切除进行亚组分析（肺段切除和楔形切除），因此其结果的可信程度受到很多学者的质疑。一些回顾性临床研究的结果显示对于因心肺功能不佳而不能接受肺叶切除的老年早期肺癌患者，亚肺叶切除术尤其是解剖性肺段切除术可以获得类似于肺叶切除的治疗效果。近年一项美国的研究^[8]^将肿瘤最大径≤2 cm并接受亚肺叶切除的NSCLC患者按年代分为早期（1988年-1998年）、中期（1999年-2004年）和近期（2004年-2008年）3组并对不同年代接受肺叶切除与亚肺叶切除的患者进行预后分析，结果显示早期组肺叶切除生存明显优于亚肺叶切除；中期组肺叶切除与肺楔形切除比较优势依然存在，但较肺段切除术已无明显优势；近期组肺叶切除术的优势则完全消失。

然而目前我们所获得的肺叶切除和亚肺叶切除效果比较的研究结果绝大部分来源于回顾性研究，研究中可能存在术前患者选择偏倚、肿瘤组织学类型和肿瘤大小等偏倚因素。目前国内外已经注册并开展的肺叶切除和亚肺叶切除效果比较的前瞻性研究中，作为最早开展的JCOG0802^[9]^和CALGB140503^[10]^研究有望在不久之后为该争论给出确切的答案。

目前亚肺叶切除相关的研究证据不管从数量还是质量上都不足以使NSCLC的临床实践发生改变，在确切答案问世之前肺叶切除仍然是NSCLC外科治疗的金标准，亚肺叶切除仍然不能取代肺叶切除成为早期NSCLC的标准术式，而应该作为NSCLC患者的“妥协性”手术方式。2015年第6版NCCN指南指出，亚肺叶切除需保证切除肺组织切缘距离病变边缘≥2 cm或切缘距离≥病变最大径，并推荐用于低肺功能或具有肺叶切除禁忌证；最大径≤2 cm的外周型结节且至少具备以下特征之一：①病理类型为原位癌；②CT提示磨玻璃样成分≥50%；③影像学随访证实肿瘤倍增时间 > 400天^[11]^。

## 4 淋巴结清扫范围与方式

系统性淋巴结清扫是早期NSCLC手术治疗重要组成部分。系统性淋巴结清扫能够提供准确的术后N分期，指导术后辅助治疗。但越来越多早期NSCLC被发现并接受外科治疗，使得外科医生对于系统性淋巴结清扫的地位提出了质疑。目前国内外已经开展其他淋巴结切除方式，如系统性淋巴结采样、叶特异性淋巴结清扫、选择性淋巴结清扫等与系统性淋巴结清扫治疗早期NSCLC效果的比较研究。美国大型前瞻性随机对照研究（ACOSOG-Z0030）结果显示，与系统性淋巴结采样相比，系统性淋巴结清扫并不能改善早期NSCLC患者的无瘤生存率、局部复发率及远处转移率^[12]^。一些回顾性研究也得到类似的结论，近年美国国家肺癌协作网络指南已经把系统性淋巴结采样纳入早期NSCLC外科治疗的外科指南^[13]^。一项较大规模的回顾性研究对370例早期NSCLC接受叶特异性淋巴结采样与系统性淋巴结采样外科治疗效果进行比较，结果显示两组局部复发率无统计学差异^[14]^。依据已有研究结果^[14, 15]^，2006年欧洲胸外科协会指南中指出叶特异性淋巴结清扫可以应用于外周型T1期鳞癌患者^[16]^。

随着国家肺筛选试验的阳性结果得到证实，高危人群肺癌筛查已经在欧美国家及我国相继开展，以磨玻璃影（ground glass opacity, GGO）为特点的早期肺癌（如非典型腺瘤样增生、原位癌、或者微浸润癌）进入了大家视野。这类早期肺癌侵袭性非常低，一般而言极少发生纵隔淋巴结转移^[17]^。有学者认为对于IA期肺腺癌中影像表现为纯GGO或只有极少实变的GGO（实变成分 < 5 mm）者，可不用进行系统性淋巴结清扫，对纯实性结节或部分实变的GGO（实变成分 > 5 mm）则建议行淋巴结清扫^[18-20]^。

基于上述结果，我们有理由认为虽然系统性淋巴结清扫目前仍是早期NSCLC外科治疗的首选，但一部分早期NSCLC患者，如非典型腺瘤样增生、原位癌及部分微浸润癌可能不会发生淋巴结转移，系统性淋巴结清扫并不能为其带来生存获益，个体化的淋巴结清扫方式可能在未来成为治疗这一部分早期NSCLC的重要组成部分。因此对NSCLC淋巴结转移规律的探索以及对不同淋巴结清扫方式效果比较的前瞻性随机对照研究有可能在未来成为改变目前淋巴结清扫方式的主要依据。
